# One‐Step Formation of Low Work‐Function, Transparent and Conductive MgF*_x_* O*_y_* Electron Extraction for Silicon Solar Cells

**DOI:** 10.1002/advs.202202400

**Published:** 2022-06-17

**Authors:** Junjun Li, Cong Guo, Yu Bai, Wenzhu Liu, Yang Chen, Jialong He, Dongdong Li, Xinbo Yang, Qingqing Qiu, Tao Chen, Junsheng Yu, Yuelong Huang, Jian Yu

**Affiliations:** ^1^ Institute of Photovoltaics Southwest Petroleum University Chengdu 610500 China; ^2^ Shanghai Institute of Microsystem and Information Technology Chinese Academy of Sciences (CAS) Shanghai 201800 China; ^3^ The Interdisciplinary Research Center Shanghai Advanced Research Institute Chinese Academy of Sciences Shanghai 201210 China; ^4^ College of Energy Soochow Institute for Energy and Materials InnovationS (SIEMIS) Soochow University Suzhou 215006 China; ^5^ State Key Laboratory of Electronic Thin Films and Integrated Devices School of Optoelectronic Information University of Electronic Science and Technology of China (UESTC) Chengdu 610054 China; ^6^ State Key Lab Oil and Gas Reservoir Geol and Exploita Southwest Petroleum University Chengdu 610500 China

**Keywords:** dopant‐free solar cells, electron selective contact, high transmittance, LiF*_x_*/MgF*_x_* O*_y_* stack, low work function

## Abstract

The development of high‐performance dopant‐free silicon solar cells is severely bottlenecked by opaque electron selective contact. In this paper, high transmittance (80.5% on glass) and low work function (2.92 eV) lithium fluoride (LiF*_x_*)/MgF*_x_* O*_y_* electron contact stack by tailoring the composition of MgF*_x_* O*_y_* hybrid film is reported. This hybrid structure exhibits a high conductivity (2978.4 S cm^−1^) and a low contact resistivity (2.0 mΩ cm^2^). The element profile of LiF*_x_*/MgF*_x_* O*_y_* contact is measured and the reaction kinetics is analyzed. As a proof‐of‐concept, this electron selective contact is applied for dopant‐free silicon solar cells. An impressive efficiency of 21.3% is achieved on dopant‐free monofacial solar cell with molybdenum oxide (MoO*_x_*)/zinc‐doped indium oxide (IZO) hole contact. An efficiency bifaciality of 71% is obtained for dopant‐free bifacial solar cell with full‐area LiF*_x_*/MgF*_x_* O*_y_*/ITO (tin‐doped indium oxide) transparent electron contact. It is the highest efficiency bifaciality so far for dopant‐free bifacial solar cells to the best knowledge. Both cell configurations with LiF*_x_*/MgF*_x_* O*_y_* contacts show excellent environment stability. The cell efficiency maintains more than 95% of its initial value after keeping in air for 1500 h. This work provides a new idea to achieve transparent electron contact, showing a great potential for high‐efficiency and low‐cost optoelectronic devices.

## Graphical abstract

A low work function, transparent, and conductive lithium fluoride (LiF*_x_*)/MgF*_x_* O*_y_* electron extraction stack is reported. By applying the electron contact, a dopant‐free monofacial silicon solar cell with an impressive efficiency of 21.3% is achieved. And an efficiency bifaciality of 71% is reported for bifacial device. This work provides a new idea to achieve transparent electron selective contacts.

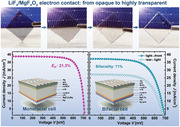

## 1 Introduction

Silicon heterojunction (SHJ) solar cells have attracted great attention due to high efficiency, simple structure, and low temperature process. Benefiting from the phosphorus and boron doped hydrogenated amorphous silicon (n‐a‐Si:H and p‐a‐Si:H) layers capped on the intrinsic amorphous silicon (i‐a‐Si:H) layers, significant band bending at the interface and excellent field passivation are achieved.^[^ ^1^, ^2^, ^3^, ^4^, ^5^ ^]^ A record efficiency of 26.3% on M6 size SHJ solar cell has been reported by Longi Solar,^[^ ^6^ ^]^ where the parasitic absorption and carrier recombination is efficiently controlled. In order to reduce the parasitic absorption caused by the doped amorphous silicon layers, an increasingly popular approach of substituting the doped layers with hole and electron selective materials has been developed as dopant‐free silicon solar cells. Wide band gap and high work function (WF) molybdenum oxide (MoO*_x_*),^[^ ^7^, ^8^, ^9^, ^10^, ^11^, ^12^, ^13^, ^14^ ^]^ tungsten oxide (WO*_x_*),^[^ ^8^, ^9^, ^10^, ^11^, ^12^, ^14^, ^15^ ^]^ vanadium oxide (VO*_x_*),^[^ ^8^, ^9^, ^11^, ^12^, ^16^, ^17^ ^]^ and nickel oxide (NiO*_x_*)^[^ ^8^, ^12^, ^14^, ^18^ ^]^ have been demonstrated as the hole selective contacts. The external quantum efficiency (EQE) responses of these front contacts certify it could promote the short‐circuit current density (*J*_sc_, gain up to 1 mA cm^−2^) thanks to the lower light parasitic absorption.^[^ ^19^, ^20^ ^]^ Yet the collection of electrons in these cells is still realized by n‐a‐Si:H layers or phosphorus‐diffused back surface field.

Two‐side‐contacted dopant‐free silicon solar cells have the potential to achieve high efficiency with low‐cost procedure.^[^ ^21^, ^22^ ^]^ An efficiency of 19.4% has been obtained for the cell featuring an i‐a‐Si:H/LiF*_x_* (lithium fluoride)/Al (Aluminum) electron selective stack and MoO*_x_* hole transport layer.^[^ ^23^ ^]^ The efficiency has been further improved to 20.7% by inserting a TiO*_x_* (titanium oxide) (1.5 nm) layer between i‐a‐Si:H and LiF*_x_*/Al.^[^ ^24^ ^]^ Zhong et al. also find that a thicker ZnO (zinc oxide) film (75 nm) capped with LiF*_x_*/Al enables efficient electron selectivity and suppression of parasitic infrared absorption, leading to an efficiency of 21.4%.^[^ ^21^ ^]^ It should be noted that these impressive progresses are all based on monofacial dopant‐free solar cells. More low WF compounds such as magnesium oxide (MgO*_x_*),^[^ ^25^, ^26^, ^27^ ^]^ tantalum oxide (TaO*_x_*),^[^ ^28^ ^]^ tantalum nitride (TaN*_x_*),^[^ ^29^ ^]^ titanium nitride (TiN*_x_*),^[^ ^30^ ^]^ magnesium fluoride (MgF*_x_*),^[^ ^31^ ^]^ cesium carbonate (CsCO_3_),^[^ ^32^ ^]^ etc., have been used as the electron selective contact. Nevertheless, all of these materials need to combine with additional low WF metal (such as Al or (calcium) Ca/Al, etc.) to form rear side metal electrode.^[^ ^25^, ^26^, ^27^, ^28^, ^29^, ^30^, ^31^, ^32^, ^33^, ^34^, ^35^, ^36^ ^]^ However, this opaque stack is unsuitable for bifacial structure.

The further development of highly‐efficient dopant‐free bifacial solar cell is challenged by the opaque electron selective contact. Only a few groups reported the bifacial structure. A most recent work has reported on the ZnO/grid‐designed LiF*_x_*/Al electron selective contact.^[^ ^37^ ^]^ Yet the highest short current density bifaciality is only 44% with the optimized Al grid fraction, and the rear‐side efficiency is 0.7% under 0.15 sun illumination. Therefore, the design of transparent and conductive electron selective contact material is of high priority for dopant‐free bifacial solar cell. In this study, a transparent hybrid film with mixed phases of magnesium (Mg), magnesium fluoride (MgF*_x_*), and magnesium oxide (MgO*_y_*) has been developed, where the Mg layer is in situ co‐reacted by fluorine (F) and oxygen (O). We define this hybrid film as MgF*_x_* O*_y_* film. The composition of MgF*_x_* O*_y_* film is tailored by precisely controlling the deposition rate of Mg layer and the low‐temperature vacuum annealing process. The element profile, optoelectronic properties are tested and reaction kinetics is analyzed. As a proof‐of‐concept, both monofacial and bifacial dopant‐free solar cells have been fabricated with full‐area LiF*_x_*/MgF*_x_* O*_y_* electron selective contact. This work provides a new idea to achieve transparent electron selective contact with an alkali metal fluoride and alkaline earth metal stack, which shows a great potential for high efficiency and low‐cost optoelectronic devices, such as dopant‐free solar cell, perovskite solar cell, and perovskite/silicon tandem solar cell.

## 2 Results and Discussion

### 2.1 Highly Transparent, Low Work Function, and Conductive LiF*_x_*/MgF*_x_* O*_y_* Film Stack

**Figure 1:**
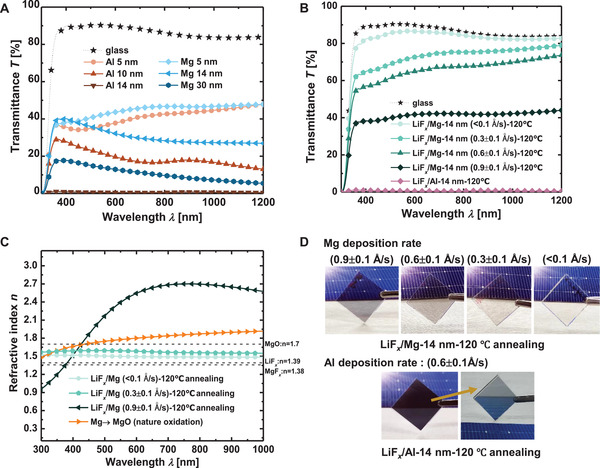
Optical transmittance spectra, refractive index, and photos of different electron contacts. A) Transmittance spectra with different thickness of Mg and Al on glass; B) transmittance spectra of LiF*_x_*/Al and LiF*_x_*/Mg on glass with different deposition rate and 120 °C post annealing treatment; C) refractive index of different Mg deposition conditions for LiF*_x_*/Mg (120 °C) and Mg; D) photos of LiF*_x_*/Mg (120 °C) contact on glass with different Mg deposition rate and LiF*_x_*/Al (120 °C) contact.

The optical properties of different electron selective contacts are compared in **Figure** **1**. Due to the metal reflection, the transmittance of metal Al and Mg decreases significantly with the increased thickness (shown in Figure 1A). The average transmittance (*T*) of Al and Mg (each the thickness is 5 nm) on glass substrate is 39.6% and 42.9% in the wavelength range of 300–1200 nm, and further decreases to 0.6% and 29.8% when the thickness is 14 nm, respectively. After inserting an ultra‐thin LiF*_x_* (0.75 nm) between metal and glass substrate, the transmittance of LiF*_x_*/Al (14 nm) stack is nearly un‐changed even after post‐annealing process. Interestingly, the transmittance of LiF*_x_*/Mg (14 nm) stack is significantly influenced under different Mg deposition rate (*R*_Mg_) and low temperature (120 °C) annealing process. The transmittance of LiF*_x_*/Mg (120 °C) with different *R*_Mg_ from 0.9 ± 0.1 to < 0.1 Å s^−1^ are also analyzed in Figure 1B. By decreasing *R*_Mg_, the average transmittance in 300–1200 nm increases significantly from 39.9% to 80.5%. The transmittance of glass substrate is 83.4% using air as baseline in this work. Figure 1D evidently observes the negative correlation between the transmittance and *R*_Mg_. As a consequence, LiF*_x_*/Mg (120 °C) transform from opaque to highly transparent. While the LiF*_x_*/Al (14 nm) stack shows a clear specular reflection. Figure 1C shows the refractive index (*n*) of LiF*_x_*/Mg (120 °C) stack and Mg (Mg changes to MgO due to natural oxidation). The deposition rate *R*_Mg_ are 0.9 ± 0.1, 0.3 ± 0.1, and <0.1 Å s^−1^, respectively. The refractive index of the LiF*_x_*/Mg (120 °C) cannot be fitted when deposition rate at 0.9 ± 0.1 Å s^−1^, which may be ascribed to the metal reflection. When *R*_Mg_ < 0.1 and 0.3 ± 0.1 Å s^−1^, the value of refractive index is between MgO (*n* = 1.7) and MgF*_x_* (*n* = 1.39). The refractive index of MgO is also presented when pure Mg is fully oxidized to MgO. According to the optical measurement results, we initially speculate that the rapidly increase transmittance is caused by the interaction between LiF*_x_* and Mg at different deposition rate and post‐treatment process, leading to the phase change. Figure S1, Supporting Information finds the 14‐nm‐thick Mg is optimal for devices in consideration of the balance between transmission and interface contact.

**Figure 2:**
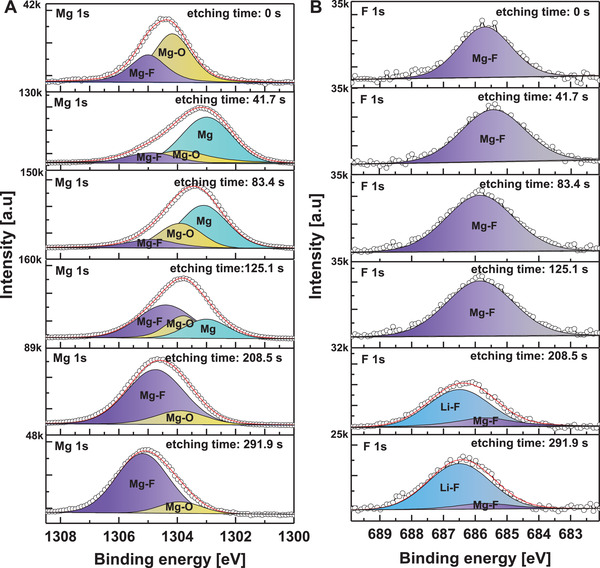
The XPS spectra and the longitudinal elemental distribution profile. A) Mg 1s core level of LiF*_x_*/Mg (120 °C) contact surface with Ar ions etching, etching time from 0 to 291.9 s; B) F 1s core level of LiF*_x_*/Mg (120 °C) contact surface with Ar ions etching, etching time from 0 to 291.9 s.

To investigate the interaction between LiF*_x_* and Mg, the chemical composition of LiF*_x_*/Mg (120 °C) stack is characterized by high‐resolution X‐ray photoelectron spectroscopy (XPS) combined the element profiling. **Figure** **2**A,B shows the XPS peaks of Mg 1s and F 1s core level spectra as a function of the surface etching time. Before surface etching, Mg 1s peak can be fitted by two peaks, the purple Mg 1s peak at 1305 eV and shallow yellow peak originate from Mg‐F^[^ ^31^ ^]^ and Mg‐O, respectively. The O presence of Mg‐O stems from the native oxidation (shown in Figure S2, Supporting Information). At the etching time of 41.7 s, the Mg 1s peak is observed at 1303 eV, and the Mg‐F and Mg‐O contents start to increase and reach the maxima at the etching time of 125.1 s, qualitatively in agreement with F 1s and O 1s. The presence of F may ascribe to the fully stoichiometric LiF*_x_* (seen in Figure S3, Supporting Information). The presence of Mg‐O could be ascribed to in situ oxidation of Mg due to the oxygen remaining in the vacuum chamber and the post‐annealing process. We also find that the oxygen in base vacuum affect the interaction between LiF*_x_*/Mg interface. The fitted XPS core level spectra in the O 1s region before and after surface etching are presented in Figure S2, Supporting Information. After 125.1 s, the Li‐F peak is detected at 686.5 eV and the content increases rapidly, while the Mg content decreases remarkably until it disappears, together with gradually reduced Mg‐O and Mg‐F peaks. The content curves of all elements with the etching time from 0 to 291.9 s are shown in Figure S4, Supporting Information. There is no obvious boundary in the LiF*_x_*/Mg stack, which causes a hybrid phase composed of Mg‐O, Mg‐F, and Mg. The existence of Mg‐F, Mg‐O, Li‐F, and Mg unambiguously proves the interchange of F, O with Mg atoms after precisely control *R*_Mg_ combined with low‐temperature and vacuum post‐annealing process, resulting in a transparent LiF*_x_*/MgF*_x_* O*_y_* film stack (shown in Figure 1B).

**Figure 3:**
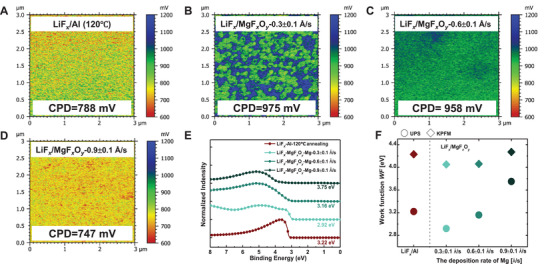
The work function measurement of different electron contacts. A) Surface potential distribution of LiF*_x_*/Al (120 °C) measured by KPFM; B–D) surface potential distribution of LiF*_x_*/MgF*_x_* O*_y_* electron contact stacks with different Mg deposition rate by KPFM, respectively; E) the UPS spectrum of LiF*_x_*/Al (120 °C) and LiF*_x_*/MgF*_x_* O*_y_* stacks with different Mg deposition rate; F) the comparison of work function (WF) measured by KPFM and UPS, respectively.

**Figure** **3**A–D shows the surface contact potential difference (CPD) of LiF*_x_*/Al (120 °C) and LiF*_x_*/MgF*_x_* O*_y_* with different *R*_Mg_ measured by Kelvin probe force microscope (KPFM). The surface potential is associated with the WF, which is determined by CPD between a Pt‐coated conductive cantilever probe and the sample surface. The detailed calculation process of WF is shown in Experimental Section. It is found that the WF of LiF*_x_*/MgF*_x_* O*_y_* at *R*_Mg_ < 0.6 ± 0.1 Å s^−1^ is lower than that LiF*_x_*/Al (120 °C), qualitatively in agreement with the ultraviolet photoelectron spectroscopy (UPS) spectrum analysis in Figure 3E. The lowest WF of LiF*_x_*/MgF*_x_* O*_y_* stack is 2.92 eV, which is 0.3 eV lower than LiF*_x_*/Al (120 °C) stack. The comparison and detailed values of WF obtained by KPFM and UPS are also shown in Figure 3F, indicating that the LiF*_x_*/MgF*_x_* O*_y_* stack has a wider process window to achieve low WF. When the deposition of Mg at a high deposition rate, Mg dominates in LiF*_x_*/MgF*_x_* O*_y_* stack, leading to the increased WF. The lower WF of LiF*_x_*/MgF*_x_* O*_y_* is anticipated to promote downward band‐bending inside the silicon wafer, extracting electrons to the surface and consequently facilitating electron collection.

Consequently, the high transmittance (80.5%) and low WF (2.92 eV) LiF*_x_*/MgF*_x_* O*_y_* electron contact stack is prepared successfully. The detailed electrical properties of LiF*_x_*/MgF*_x_* O*_y_* stack are also analyzed. The conductivity (*σ*) of LiF*_x_*/MgF*_x_* O*_y_* film stack with different *R*_Mg_ is shown in Figure S5, Supporting Information. The conductivity of LiF*_x_*/MgF*_x_* O*_y_* at *R*_Mg_ < 0.1 Å s^−1^ is close to that of pure MgF*_x_*. The conductivity increases from about 1.8 × 10^−5^ to 3 × 10^3^ S cm^−1^ as *R*_Mg_ increases from 0.15 to 0.9 ± 0.1 Å s^−1^, exhibiting adjustable conductivity. The highest conductivity of LiF*_x_*/MgF*_x_* O*_y_* could be ascribed to the larger proportion of Mg in MgF*_x_* O*_y_* films, and the lowest conductivity value may be due to majority of MgF*_x_* and MgO*_y_* in the MgF*_x_* O*_y_* film.

**Figure 4:**
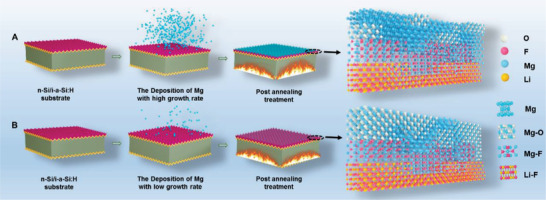
The schematic diagrams of the reaction kinetics with different Mg deposition rate. A) High Mg deposition rate; B) low Mg deposition rate.

The contact resistivity (*ρ_c_*) of LiF*_x_*/MgF*_x_* O*_y_* with different *R*_Mg_ and structures is also investigated. The Cox and Strack and transfer length method (TLM) is presented in Figure S6, Supporting Information. The contact resistance between LiF*_x_*/MgF*_x_* O*_y_* stack and i‐a‐Si:H exhibits the highest *ρ_c_* (≈600 mΩ cm^2^ average) when *R*_Mg_ < 0.1 Å s^−1^. The *ρ_c_* decreases sharply to 22.4 mΩ cm^2^ with increasing deposition rate to 0.9 ± 0.1 Å s^−1^. The thicker Mg film exhibits the lower contact resistance. The lowest conductivity of 2.0 mΩ cm^2^ between LiF*_x_*/MgF*_x_* O*_y_* and n‐Si/i‐a‐Si:H was achieved at high *R*_Mg_ with 70 nm Mg. Combining the carrier transport mechanism of heterojunction structure and the conductivity results in Figure S5, Supporting Information, we find the tunneling effect plays the significant role in contact resistance. When *R*_Mg_ at a low level, MgF*_x_* and MgO*_y_* dominate in the LiF*_x_*/MgF*_x_* O*_y_* stack. That is to say, the higher tunneling resistance and more difficult carrier transport. This phenomenon is remarkably improved at higher deposition rates, where Mg in the LiF*_x_*/MgF*_x_* O*_y_* stack promotes carrier transport. The contact resistance between LiF*_x_*/MgF*_x_* O*_y_* and Ag electrode interface, LiF*_x_*/MgF*_x_* O*_y_* and ITO interface are also calculated. Similar conclusions could be obtained. Based on aforementioned analysis, the potential interaction process between Mg, O, and F elements is illustrated in **Figure** **4**. At high *R*_Mg_, there is not enough time for atoms exchange for F and O with Mg, causing dominant Mg metal phase with high conductivity and low transmittance as shown in Figure 1B. At low *R*_Mg_, the atom interaction time is more sufficient, which results in the formation of MgF*_x_* and MgO*_y_* dominated LiF*_x_*/MgF*_x_* O*_y_* film with high transmittance. The vacuum annealing process further promotes the improvement of the interface between passivation layer and electron contact, and the stability of MgF*_x_* O*_y_* hybrid film. The influence of post annealing process on surface morphology and device performance is shown in Figures S7,S8, Supporting Information.

### 2.2 Dopant‐Free Silicon Solar Cells with LiF*_x_*/MgF*_x_* O*_y_* Electron Contact

**Figure 5:**
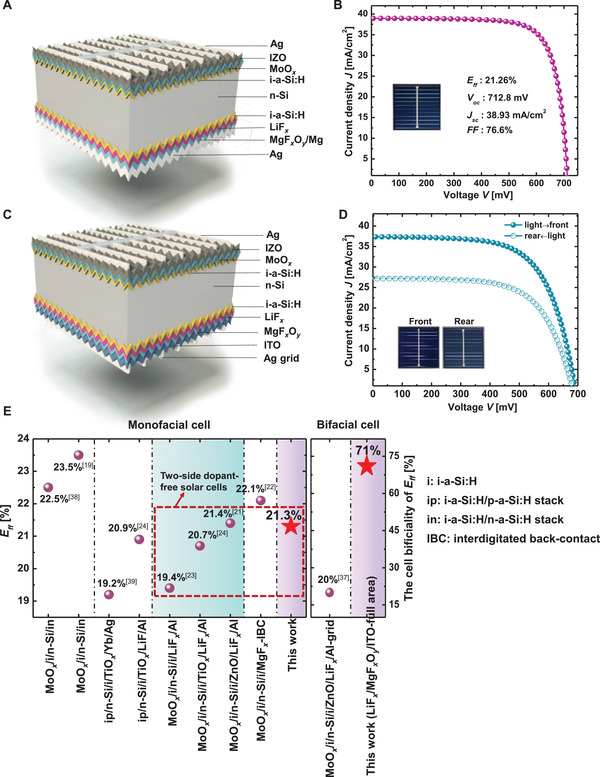
Performance of dopant‐free silicon solar cells with LiF*_x_*/MgF*_x_* O*_y_* electron contacts. A) Schematic of dopant‐free monofacial solar cell. MoO*_x_*/IZO and LiF*_x_*/MgF*_x_* O*_y_*/Mg are deposited as the hole‐selective and electron‐selective contacts, respectively; B) the *J–V* curve of monofacial device with an active area of 3.52 cm^2^; C) schematic of dopant‐free bifacial solar cell. MoO*_x_*/IZO and LiF*_x_*/MgF*_x_* O*_y_*/ITO are deposited as the hole‐selective and electron‐selective contacts, respectively; D) the *J–V* curves of bifacial solar cell with an active area of 3.52 cm^2^; E) comparison of cell efficiency (*E*_ff_) in this work with reported dopant‐free carrier selective contact stacks.

As a proof‐of‐concept, both dopant‐free monofacial and bifacial silicon solar cells with full‐area LiF*_x_*/MgF*_x_* O*_y_* transparent electron selective contacts were fabricated. The cell structures and electrical performance are shown in **Figure** **5**. The hole extraction layer at the front side consists of an i‐a‐Si:H passivation layer, MoO*_x_* film (WF = 5.93 eV, shown in Figure S9, Supporting Information) and an IZO film. For monofacial structure, the LiF*_x_*/MgF*_x_* O*_y_* stack is prepared at a *R*_Mg_ of 0.9 ± 0.1 Å s^−1^ and 70 nm‐thick Mg layer. A champion efficiency of 21.3% has been achieved, with *V*_oc_ of 712.8 mV, *J*_sc_ of 38.93 mA cm^−2^_,_ and FF of 76.6%, respectively. This high electrical performance proves the LiF*_x_*/MgF*_x_* O*_y_* stack has effective electron selective transport. Meanwhile, the full‐area and transparent LiF*_x_*/MgF*_x_* O*_y_*/ITO stack is employed for dopant‐free bifacial solar cells. The bifacial solar cell shows a front *E*_ff_ of 16.9% with *V*_oc_ of 691.6 mV, *J*_sc_ of 37.4 mA cm^−2^, and FF of 65.3%. An efficiency bifaciality of 71% is calculated at a rear side efficiency of 12.0% (detailed results shown in Table S1, Supporting Information). The relatively low *J*_sc_ could be ascribed to the decreased transmittance of LiF*_x_*/MgF*_x_* O*_y_* stack which needs to compromise the lateral conductivity. It should be noted that the FF of bifacial structure is obviously lower than monofacial structure, which could be ascribed to the deteriorated lateral conductivity and increased contact resistance of LiF*_x_*/MgF*_x_* O*_y_* stack at relatively low *R*_Mg_. We have compared the cell efficiency in this work with the reported monofacial and bifacial silicon solar cells featuring carrier selective contact stack in Figure 5E. An impressive efficiency has been presented on monofacial structure. The dopant‐free bifacial silicon solar cell has an enhanced bifaciality from ≈20% to 70%, compared to the most recent publication using ZnO/grid‐designed LiF*_x_*/Al electron selective contact.^[^ ^37^ ^]^ The EQE and the calculated photogenerated current density are also presented from spectral response (shown in Figure S10, Supporting Information). Compared with EQE results of SHJ solar cell with an impressive efficiency of 24.3%, the spectral response of dopant‐free solar cell in this work is obviously lower at 300–500 nm and 800–1100 nm wavelength band, resulting in a 0.28 and 1.02 mA cm^−2^ photogenerated current density loss, respectively. Detailed performance optimization is ongoing.

**Figure 6:**
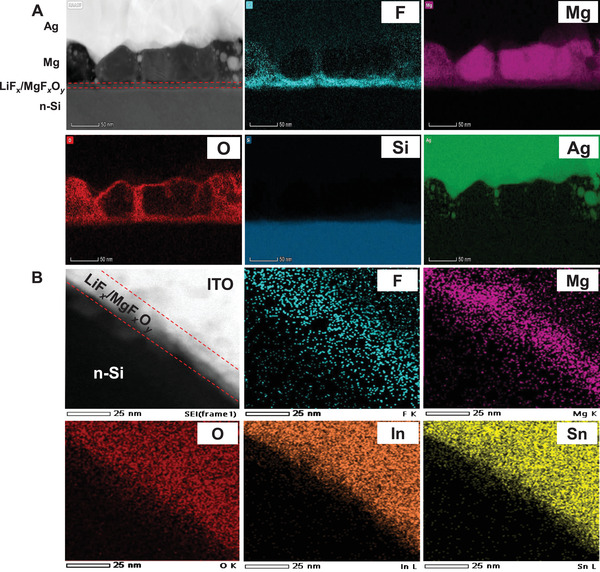
STEM microscopy images and EDX mapping of dopant‐free solar cells. A) Dopant‐free monofacial solar cell with n‐Si/i‐a‐Si:H/LiF*_x_*/MgF*_x_* O*_y_*/Mg/Ag contact stack; B) dopant‐free bifacial solar cell with n‐Si/i‐a‐Si:H/LiF*_x_*/MgF*_x_* O*_y_*/ITO contact stack.

The cross section and microstructure of both monofacial and bifacial structure with LiF*_x_*/MgF*_x_* O*_y_* electron contact is also analyzed via high‐angle annular dark‐field (HAADF) images by scanning transmission electron microscopy (STEM), and the energy‐dispersive X‐ray spectra (EDX). The small atomic weight of Li is difficult to detect by EDX and makes it susceptible to severe knock‐on effects by the electron beam. Therefore, the F signal is detected as the evolution process. The dopant‐free monofacial solar cell with LiF*_x_*/MgF*_x_* O*_y_* as electron contact is shown in **Figure** **6**A. It can be found that Mg, F, and O signals on the n‐Si/i‐a‐Si:H surface don't have an obvious boundary, which is consistent with the results of the XPS longitudinal element distribution shown in Figure 2. The existence of O might be attributed to in situ oxidation during post‐annealing. Note that the Mg is thicker than bifacial device to achieve better electrical performance. For dopant‐free bifacial device with LiF*_x_*/MgF*_x_* O*_y_*/ITO as electron contact, it should be noted that the distribution of EDX signal of F, Mg, and O is basically the same as that of monofacial structure shown in Figure 6B. The distribution of these signals clearly indicates the interaction of F, O with Mg, forming a transparent LiF*_x_*/MgF*_x_* O*_y_* film stack. The EDX signals of O, In, and Sn is attributed to ITO film. It can be noted that a small amount of F diffuses into the ITO layer, which is likely to be another factor affecting the electrical property of the bifacial device.

**Figure 7:**
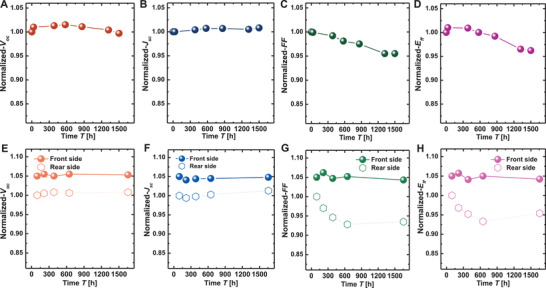
The stability of dopant‐free solar cells stored in air atmosphere at room temperature. A–D) Normalized optical and electrical performance changes of *V*_oc_, *J*_sc_, FF, and *E*_ff_ for dopant‐free monofacial solar cell; E–H) normalized optical and electrical performance changes of *V*_oc_, *J*_sc_, FF, and *E*_ff_ for dopant‐free bifacial solar cell, under front and rear side illumination, respectively.

The normalized long‐term reliability of both monofacial and bifacial cells are evaluated, as shown in **Figure** **7**. For the monofacial device, the *V*_oc_ has a slight increase (about 10 mV) at the first hundreds of hours and remains stable during the subsequent 1500 h, indicating excellent and stable passivation and electron selectivity. The degradation of *J*_sc_ is negligible in 1500 h environmental stress. The *E*_ff_ maintained more than 96% of its initial value after storage in air for 1500 h mainly attribute to the slight degradation of FF. The dopant‐free silicon solar cells with LiF*_x_*/MgF*_x_* O*_y_* contact exhibit excellent environmental stability, which is significantly improved comparing to the reported results using ZnO/LiF*_x_*/Al electron contact (93% open‐circuit voltage and 88% fill factor retention rate in air after 380 h).^[^ ^40^ ^]^ The similar evolution trend can be found for the bifacial device, shown in Figure 7E–H (the normalized data of front side properties is uniformly shifted upward by 5% for clear description). The rear‐side *E*_ff_ still maintained 95.8% of its initial value after storage in air for 1500 h. It should be noted that the rear‐side FF of bifacial cell drops faster than that of monofacial cells, which may be attributed to i) the deterioration of LiF*_x_*/MgF*_x_* O*_y_* lateral conductivity after keeping in air due to the further interchange of F, O with Mg, ii) a reduction of the contact resistivity between LiF*_x_*/MgF*_x_* O*_y_* and ITO at relatively low *R*_Mg_, and iii) diffusion of F into the ITO film as observed STEM images in Figure 6B, which might result in the deterioration of ITO conductivity.

## 3 Conclusions

In this work, we report low WF (2.92 eV) and transparent (80.5% on glass) LiF*_x_*/MgF*_x_* O*_y_* electron selective contact. The interaction evolution of F, O with Mg atoms leads to a metal phase transition and results in a transparent MgF*_x_* O*_y_* film. This hybrid structure exhibits a high lateral conductivity (up to 2978.4 S cm^−1^) and a low contact resistance (low to 2.0 mΩ cm^2^). As a proof‐of‐concept, both monofacial and bifacial structure dopant‐free silicon solar cells are fabricated. An impressive efficiency of 21.3% on monofacial structure and an efficiency bifaciality of 71% on bifacial structure have been achieved. Both devices exhibit excellent environment stability after keeping in air for 1500 h. This work provides a new cost‐effective method to prepare transparent and conductive MgF*_x_* O*_y_* films with low WF in one deposition step. The MgF*_x_* O*_y_* films show a great potential for its application in high efficiency and low‐cost optoelectronic devices, such as dopant‐free solar cell, perovskite solar cell and perovskite/silicon tandem solar cell.

## 4 Experimental Section

### Preparation of LiF*_x_*/MgF*_x_* O*_y_* Film Stack

A LiF*_x_* thin film with the thickness of 0.75 nm was deposited from LiF*_x_* particles (99.99%) by thermal evaporation at room temperature with a growth rate 0.1 Å s^−1^ and ≈5 × 10^−4 ^Pa base pressure, followed by thermal evaporated 14 nm‐thick Mg from Mg source (99.995%) on LiF*_x_* layer at a base pressure of ≈6.5 × 10^−4 ^Pa with different growth rate (from <0.1 to 0.9 ± 0.1 Å s^−1^). Then, LiF*_x_*/Mg stack was post‐annealed for 90 min at 120 °C under a varied vacuum from 10^−4^ to 10^−1^ Pa. The LiF*_x_*/Mg became transparent LiF*_x_*/MgF*_x_* O*_y_* hybrid film stack and the composition of MgF*_x_* O*_y_* film was determined by controlling Mg deposition rate, base pressure, and post‐annealing processes. Finally, LiF*_x_*/MgF*_x_* O*_y_* film stacks with different photoelectrical properties were obtained.

### Preparation of Dopant‐Free Silicon Solar Cells

N‐type FZ c‐Si (100) wafers with a resistivity of 1.0–5.0 Ω.cm and thickness of 170 µm were immersed in a 4.5 wt% KOH solution at 80 °C for 20 min to remove the saw damage and form a random pyramid surface texture. The intrinsic amorphous (i‐a‐Si:H) layers (5 nm) were deposited on both sides of textured wafers by plasma‐enhanced chemical vapor deposition (13.56 MHz). For monofacial device rear contact, 0.75 nm LiF*_x_* film was deposited on n‐Si/i‐a‐Si:H substrate, followed 70 nm‐thick Mg on LiF*_x_* layer with a growth rate 0.9 ± 0.1 Å s^−1^. Subsequently, full‐area Ag rear electrode with a thickness of 350 nm was deposited by thermal evaporation. For bifacial device, a LiF*_x_*/MgF*_x_* O*_y_* stack was prepared at an Mg deposition rate of around 0.3 ± 0.1 Å s^−1^ to obtain transparent electron contact stack, 80 nm ITO film was prepared by DC magnetron sputtering from a ITO target consisting of In_2_O_3_:SnO_2_ with 90/10 wt% ratio with a power (90 W) and a working pressure (0.5 Pa) at a base pressure of ≈5 × 10^−4^ Pa. It should be also noted that the LiF*_x_*/MgF*_x_* O*_y_* stack was post‐annealed for 90 min at 120 °C under a varied vacuum of 10^−4^ to 10^−1^ Pa for both monofacial and bifacial solar cells. Finally, 350 nm‐thick Ag grid electrodes were evaporated on rear side through a shadow mask to achieve bifacial dopant‐free solar cell.

For the front contact, 7 nm MoO*_x_* film was deposited by thermal evaporation from a MoO_3_ source (99.99%) at room temperature with a growth rate 0.1 Å s^−1^ at a base pressure of ≈6 × 10^−4^ Pa. IZO film with the thickness of 80 nm was deposited on MoO*_x_* layer by RF magnetron sputtering from an IZO target (In_2_O_3_:ZnO with 90/10 wt%) with a power (100 W) and a working pressure (0.5 Pa) at a base pressure of ≈5 × 10^−4^ Pa. Finally, 350 nm thick Ag front grid electrode was deposited onto the IZO layer through a shadow mask (active area 3.52 cm^2^).

### Contact Measurement

Contact resistivity *ρ_c_* of three contact structures (n‐Si/i‐a‐Si:H and LiF*_x_*/MgF*_x_* O*_y_*, n‐Si/i‐a‐Si:H/LiF*_x_*/MgF*_x_* O*_y_* and Ag, and n‐Si/i‐a‐Si:H/LiF*_x_*/MgF*_x_* O*_y_* and ITO/Ag) were measured by using the Cox and Strack and TLM.^[^ ^41^, ^42^ ^]^ The LiF*_x_*/MgF*_x_* O*_y_* contact (LiF*_x_* = 0.75 nm, Mg = 14 or 70 nm, covering layer Ag = 350 nm, respectively) were fabricated with different Mg deposition rate from <0.1 to 0.9 ± 0.1 Å s^−1^. Each TLM set was isolated along its edges to confine the current, the contact resistivity *ρ_c_* was extracted by fitting the trend of resistance versus spacing of the rear contacts. Full layers 70 nm Mg and 350 nm Ag were deposited on one side of the i‐a‐Si:H/n‐Si/i‐a‐Si:H by thermal evaporation to form ohmic contact as shown in Figure S1, Supporting Information. Mg with different thickness from 0 to 70 nm were deposited on the other side through a shadow mask to define contact properties by dark *I–V* measurement.

### Characterization of Film Stacks

The optical transmittance of Al, Mg, LiF*_x_*/Al (120 °C annealing), and LiF*_x_*/Mg (120 °C annealing) with different metal (Al and Mg) deposition conditions on glass substrate was examined by using UV–vis–NIR spectrophotometer (UV‐3600 plus) with a BaSO_4_ integrating sphere using air as baseline. The refractive index of LiF*_x_*/Mg (120 °C annealing) with different Mg deposition rate and naturally oxidized pure Mg was measured by ellipsometer. The longitudinal element distribution profile of LiF*_x_*/MgF*_x_* O*_y_* was detected by XPS (Thermo Scientific K‐Alpha+) with Ar ions etching (from 0 to 291.9 s). The KPFM^[^ ^43^ ^]^ (KEYSIGHT Technologies 7500) measurements were performed to obtain surface potential of LiF*_x_*/Al (120 °C annealing), LiF*_x_*/MgF*_x_* O*_y_* stacks. The surface potential was associated with the WF, which was determined by CPD between a Pt‐coated conductive cantilever probe and the samples. The WF of Pt‐coated tip and sample can be calculated using Equation (1).

(1) $$\mathrm{CPD}=(\Phi_{\mathrm{tip}}-\Phi_{\mathrm{sample}})/e$$

where *Ф*_tip_ is WF of the tip, *Ф*_sample_ is WF of the sample surface, and e is the elementary charge of electron. Herein the WF of the Pt/Cr‐coated tip was calibrated with the HOPG sample (*Ф*_HOPG_ = 4.6 eV, CPD = 420 mV). Therefore, the WF of *Ф*_tip_ was 5.02 eV. The WF of *Ф*_sample_ can be calculated using Equation (2):

(2) $$\Phi_{\mathrm{sample}}=\Phi_{\mathrm{tip}}-\mathrm{eCP}D_{\mathrm{sample}}$$

The LiF*_x_*/Al (120 °C annealing) with 10 nm Al over‐layer and LiF*_x_*/MgF*_x_* O*_y_* stacks (the thickness of Mg was 14 nm) for KPFM and UPS (at a bias of −5 V, ESCALAB 250Xi) analysis were fabricated on mechanically polished, n‐type, float zone wafers, obtaining the WF. The conductivity of LiF*_x_*/MgF*_x_* O*_y_* stacks with different Mg deposition rate on a glass substrate was measured by home‐made two‐probe instrument (2450 SourceMeter) at room temperature.

### Device Characterization

The cross section images of monofacial cell with LiF*_x_*/MgF*_x_* O*_y_*/Mg electron contact and bifacial device with full‐area LiF*_x_*/MgF*_x_* O*_y_*/ITO electron contact was analyzed by transmission electron microscopy (TEM), HAADF‐STEM, and EDX). The photoelectrical performance of dopant‐free silicon solar cells was characterized by measuring the current–voltage (*I–V*) characteristics under 100 mW cm^−2^ illumination light source (Wavelab Sinus‐220, Class AAA light source) and 25 °C.

### Statistical Analysis

The performance changes of monofacial and bifacial cells stored in air atmosphere at room temperature were normalized by origin 9.1 software, using “divided by preference cell” model as normalize method, the preference cell was the initial value of *V*_oc_, *J*_sc_, FF, and *E*_ff_, respectively. The normalized *J–V* parameters of bifacial cell front side were uniformly shifted upward by 5% for distinguishing with bifacial cells. The formula *Φ = hν − E*_Cutoff_ *+* 5 V (bias voltage: −5V) was used to process the raw data measured by UPS to obtain the WF of samples, where *Φ* is WF of samples, *hν* is the energy of HeI light source (21.22 eV), secondary cutoff edge (*E*_Cutoff_) can be obtained by linear fitting of UPS spectrum. According to the analysis of XPS and TEM results, the schematic diagrams were drawn using Cinema 4D R19 software to analyze the possible reaction kinetics of MgF*_x_* O*_y_* hybrid film.

## Conflict of Interest

The authors declare no conflict of interest.

## Supporting information

Supporting Information

## Data Availability

The data that support the findings of this study are available in the supplementary material of this article.
