# Supplementary material for: One‐Step Formation of Low Work‐Function, Transparent and Conductive MgF x O y Electron Extraction for Silicon Solar Cells
Source: Adv Sci (Weinh). 2022 Jun 17;9(23):2202400. doi: 10.1002/advs.202202400 (PMC9376844; doi:10.1002/advs.202202400)
Supplement: Supplementary file 1 — Supporting Information [file ADVS-9-2202400-s001.pdf]

Supporting Information

One-step Formation of Low Work-function, Transparent and Conductive MgF_xO_y Electron Extraction for Silicon Solar Cells

Junjun Li, Cong Guo, Yu Bai, Wenzhu Liu[**] *Yang Chen, Jialong He, Dongdong Li, Xinbo Yang, Qingqing Qiu, Tao Chen, Junsheng Yu, Yuelong Huang*[***] *Jian Yu*[*]

J. Li, C. Guo, Y. Bai, Y. Chen, J. He, Q. Qiu, T. Chen, Y. Huang, J. Yu

Institute of Photovoltaics, Southwest Petroleum University, Chengdu 610500, China

E-mail: jianyu@swpu.edu.cn (J. Yu); y.huang@swpu.edu.cn (Y. Huang)

W. Liu

Shanghai Institute of Microsystem and Information Technology, Chinese Academy of Sciences (CAS), Shanghai 201800, China

E-mail: wenzhu.liu@mail.sim.ac.cn (W. Liu)

Y. Huang, J. Yu

State Key Lab Oil & Gas Reservoir Geol & Exploita, Southwest Petroleum University, Chengdu 610500, China

J-S. Yu

State Key Laboratory of Electronic Thin Films and Integrated Devices, School of Optoelectronic Information, University of Electronic Science and Technology of China (UESTC), Chengdu 610054, China

D. Li

The Interdisciplinary Research Center, Shanghai Advanced Research Institute, Chinese Academy of Sciences, Shanghai 201210, China

X. Yang

College of Energy, Soochow Institute for Energy and Materials InnovationS (SIEMIS) Soochow University, Suzhou 215006, China

TEXT

Li and co-workers reported a high transmittance (80.5% on glass) and low work function (2.92 eV) lithium fluoride (LiF_x)/MgF_xO_y electron contact stack by tailoring the composition of MgF_xO_y hybrid film, exhibiting a high conductivity (2978.4 S/cm) and a low contact resistivity (2.0 $m\Omega \cdot cm^2$). By applying the high-performance LiF_x/MgF_xO_y electron contact, a dopant-free monofacial solar cell with an impressive efficiency of 21.3% is achieved. And an outstanding efficiency bifaciality of 71% is achieved for dopant-free bifacial solar cell. This work provides a new idea to achieve transparent electron contact, showing a great potential for high-efficiency and low-cost optoelectronic devices.

Succinct text and the necessary figures, figure captions and tables related to this paper are presented in below:

Supporting Information

Part 1: Figures in the manuscript

Figure 1. Optical transmittance spectra, refractive index and photos of different electron contacts

(A) Transmittance spectra with different thickness of Mg and Al on glass; (B) Transmittance spectra of LiF_x/Al and LiF_x/Mg on glass with different deposition rate and 120 °C post annealing treatment; (C) Refractive index of different Mg deposition conditions for LiF_x/Mg (120 °C) and Mg; (D) Photos of LiF_x/Mg (120 °C) contact on glass with different Mg deposition rate and LiF_x/Al (120 °C) contact.

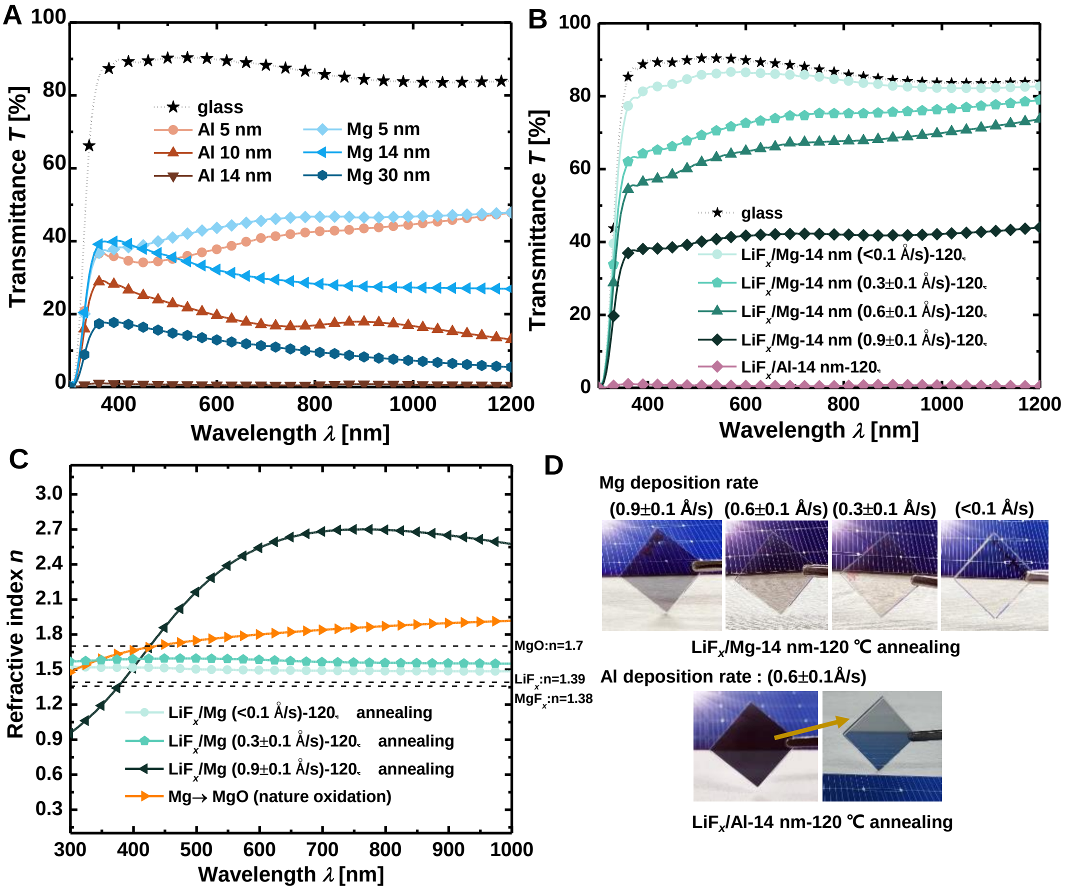

Figure 2. The XPS spectra and the longitudinal elemental distribution profile

(A) Mg 1s core level of LiF_x/Mg (120 ºC) contact surface with Ar ions etching, etching time from 0 s-291.9 s; (B) F 1s core level of LiF_x/Mg (120 ºC) contact surface with Ar ions etching, etching time from 0 s-291.9 s.

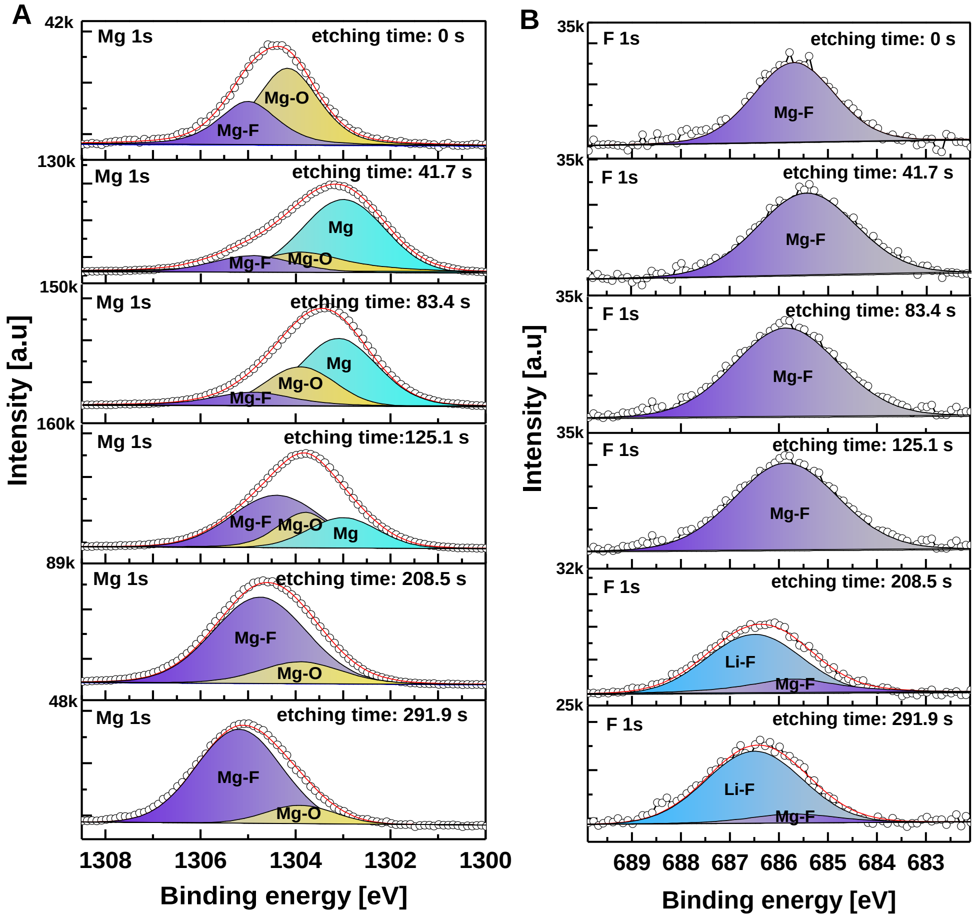

Figure 3. The work function measurement of different electron contacts

(A) Surface potential distribution of LiF_x/Al (120 ºC) measured by KPFM; (B), (C) and (D) Surface potential distribution of LiF_x/MgF_xO_y electron contact stacks with different Mg deposition rate by KPFM, respectively; (E) The UPS spectrum of LiF_x/Al (120 ºC) and LiF_x/MgF_xO_y stacks with different Mg deposition rate; (F) The comparison of work function (*WF*) measured by KPFM and UPS respectively.

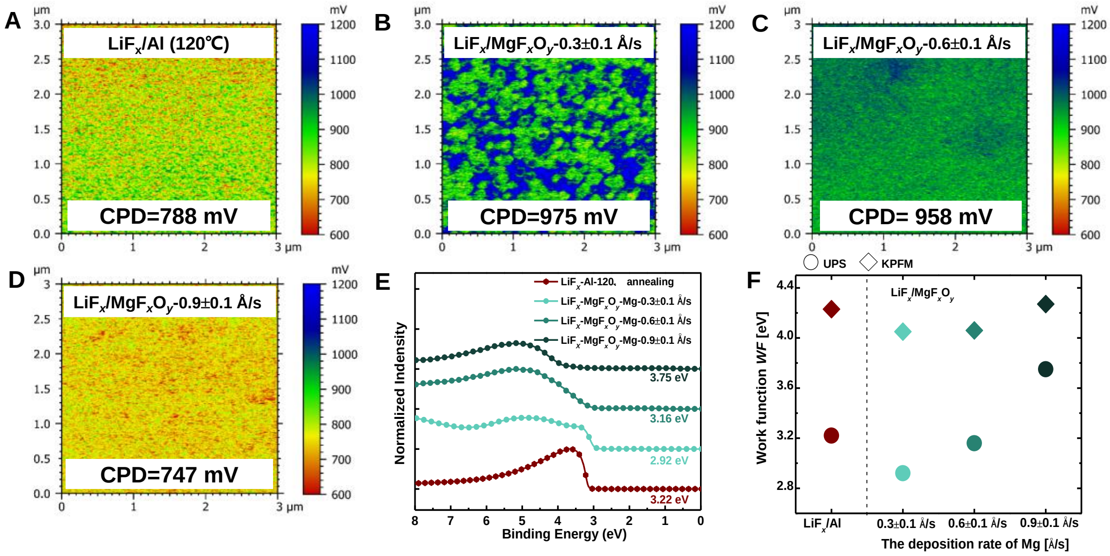

Figure 4. The schematic diagrams of the reaction kinetics with different Mg deposition rate

(A) high Mg deposition rate; (B) low Mg deposition rate.

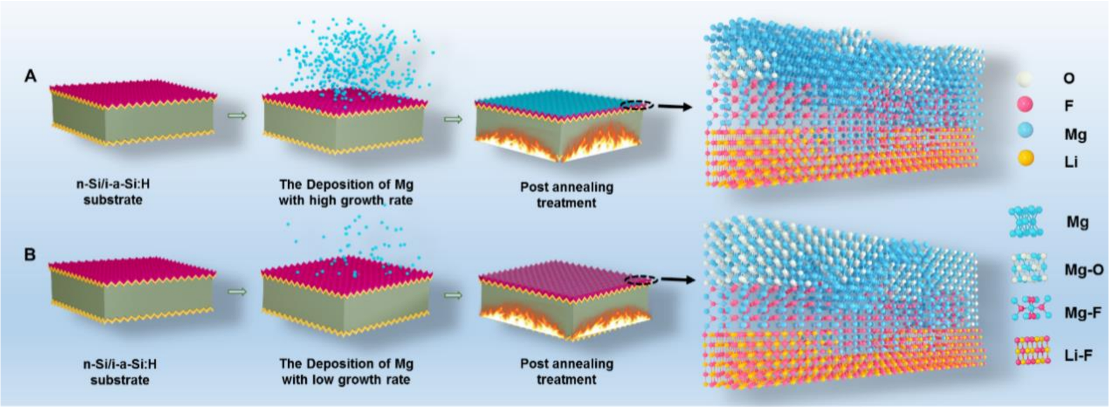

Figure 5. Performance of dopant-free silicon solar cells with LiF_x/MgF_xO_y electron contacts

(A) Schematic of dopant-free monofacial solar cell. MoO_x/IZO and LiF_x/MgF_xO_y/Mg are deposited as the hole-selective and electron-selective contacts, respectively; (B) The *J*-*V* curve of monofacial device with an active area of 3.52 cm^2; (C) Schematic of dopant-free bifacial solar cell. MoO_x/IZO and LiF_x/MgF_xO_y/ITO are deposited as the hole-selective and electron-selective contacts, respectively; (D) The *J*-*V* curves of bifacial solar cell with an active area of 3.52 cm^2; (E) Comparison of cell efficiency (E_{ff}) in this work with reported dopant-free carrier selective contact stacks.

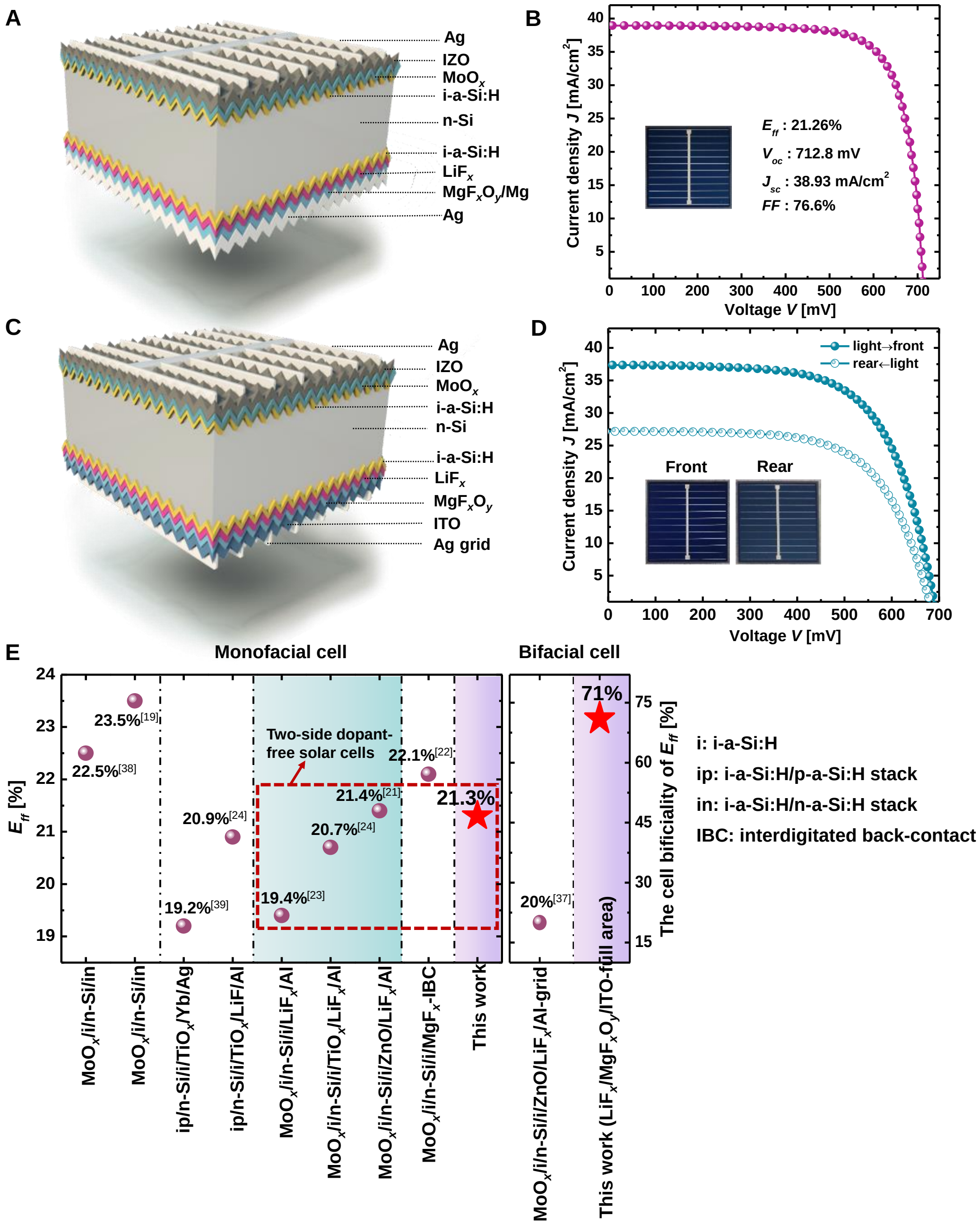

Figure 6. STEM microscopy images and EDX mapping of dopant-free solar cells

(A) Dopant-free monofacial solar cell with n-Si/i-a-Si:H/LiF$_x$/MgF$_x$O$_y$/Mg/Ag contact stack; (B) Dopant-free bifacial solar cell with n-Si/i-a-Si:H/LiF$_x$/MgF$_x$O$_y$/ITO contact stack.

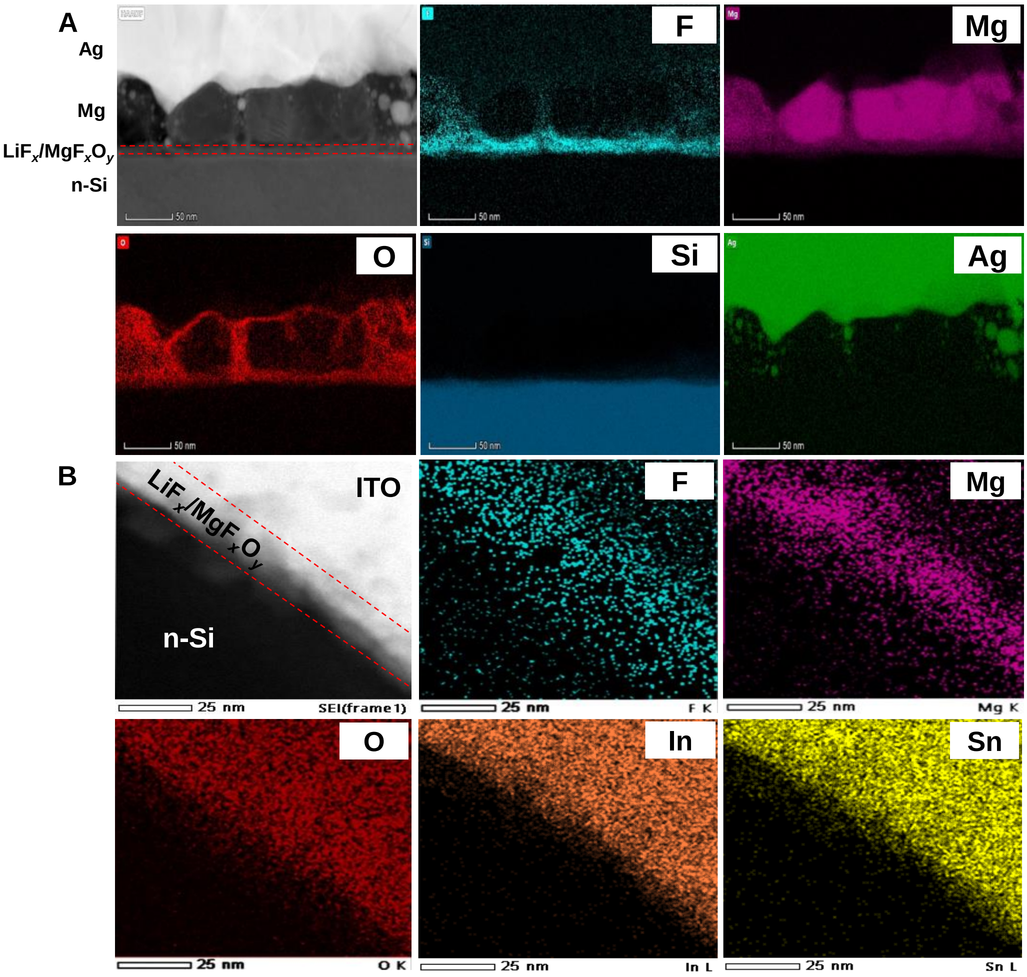

Figure 7. The stability of dopant-free solar cells stored in air atmosphere at room temperature.

(A)-(D) Normalized optical and electrical performance changes of V_{oc}, J_{sc}, FF and E_{ff} for dopant-free monofacial solar cell; (E)-(H) Normalized optical and electrical performance changes of V_{oc}, J_{sc}, FF and E_{ff} for dopant-free bifacial solar cell, under front and rear side illumination, respectively.

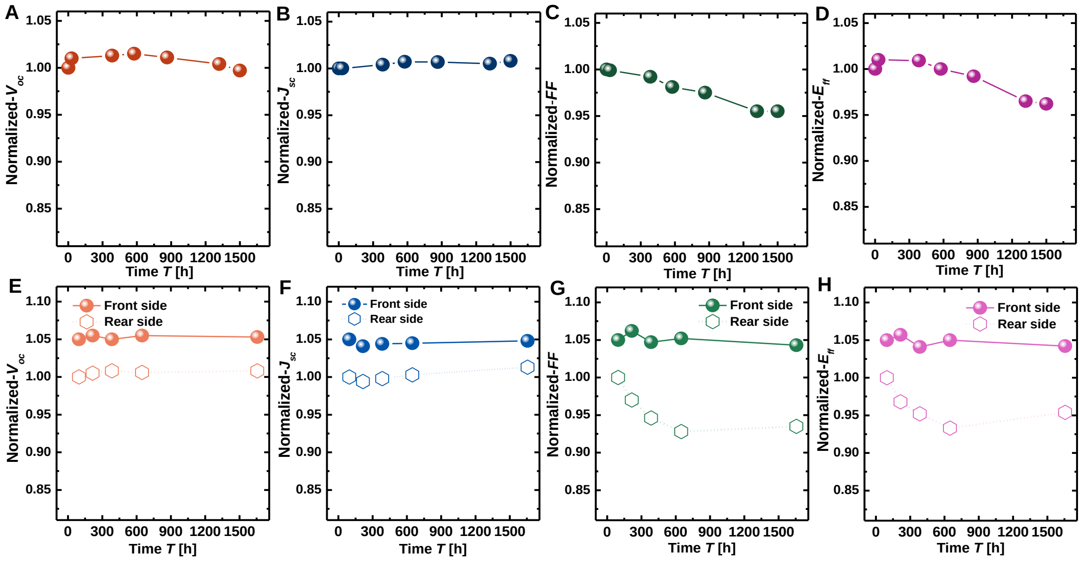

Part 2: Figures and tables in the supporting document

Figure S1:

Contact Measurement: Full layers 70 nm Mg and 350 nm Ag was deposited on front side of the i-a-Si:H/n-Si/i-a-Si:H by thermal evaporation to form ohmic contact as shown in Figure S1. Mg with different thickness from 0 to 70 nm was deposited on the rear side through a shadow mask to define contact properties by dark *I-V* measurement.

The reverse current density increases gradually with increasing Mg thickness, implying the improved contact characteristics. The anti-barrier layer with high electrical conductivity is formed at the interface between i-a-Si:H and electrode Ag with increasing Mg thickness from 10 nm to 70 nm, leading to improved contact properties. According to the surface morphology, the Mg layer with 14 nm thickness is considered to be an optimum for low resistance requirements.

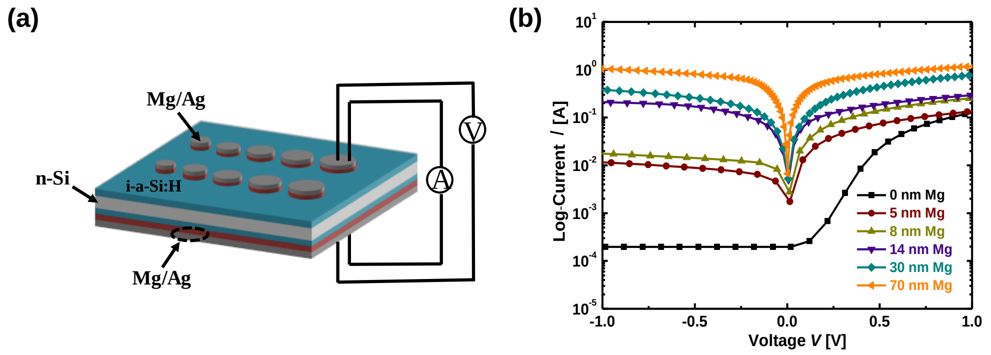

Figure S1. (a) The measured structures (pad with diameters are 1, 1.5, 2, 2.5, and 3 mm, respectively); (b) Dark *I-V* curves with Log (I) of different Mg thickness on n-Si/i-a-Si:H substrate.

Figure S2:

Characterization of LiF$_x$/Mg (120 °C annealing) film stacks: The longitudinal element distribution profile of LiF$_x$/Mg (120 °C annealing) was detected by X-ray photoelectron spectroscopy (XPS, Thermo Scientific K-Alpha+) with Ar ions etching (from 0 s - 291.9 s). The O 1s core level of LiF$_x$/Mg (120 °C annealing) surface also was analyzed to further vertify the formation of hybrid film stacks**.**

Before surface etching, the O peak is mainly attributed to the surface-absorbed oxygen. At the etching time of 41.7 s, the Mg-O peak are observed at about 530 eV and started to increase and reach the maximum value at 125.1 s, qualitatively in agreement with F 1s and Mg 1s core level. The presence of O could be ascribed to in-situ oxidation of Mg during the post-annealing process. After 125.1 s, Si-O and Li-O are detected and the contents increases rapidly, while the Mg-O content decrease remarkably. The Si and O may be ascribed to the n-Si/i-a-Si:H substrate and thin LiF$_x$ layer, respectively.

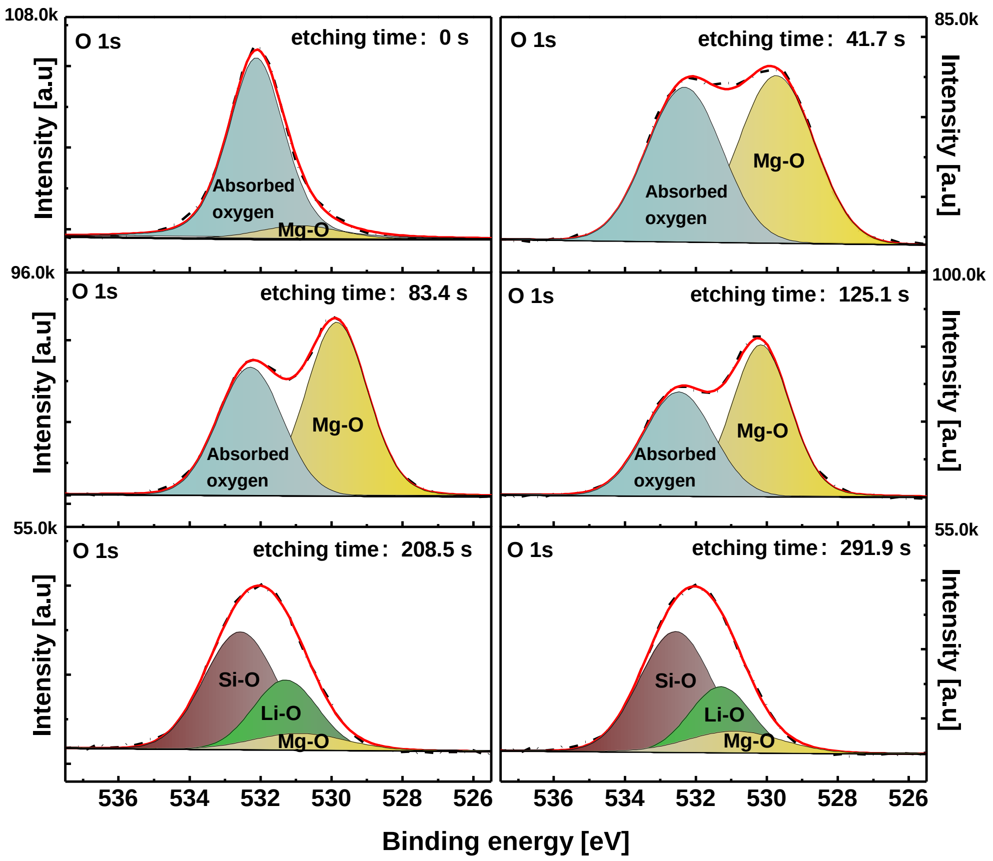

Figure S2. The O 1s core level of LiF_x/Mg (120 ^{o}C annealing) surface measured by XPS. The longitudinal elemental distribution profile is measured by Ar ions etching, with the etching time from 0 s-291.9 s.

Figure S3:

Preparation of LiF_x: LiF_x film with the thickness of 70 nm is deposited on glass substrate to measure chemical composition of LiF_x from LiF_x particles (99.99%) by thermal evaporation at room temperature with a growth rate 0.1 Å/s and ~ 6×10^{-4} Pa base pressure. *Characterization of LiF_x:* The chemical composition of LiF_x film was detected by X-ray photoelectron spectroscopy (XPS).

We estimated the stoichiometry of LiF_x film by the peak area and sensitive factor (SF) of F and Li elements, the x can be calculated using the following formula:

$$\frac{the\ peak\ area\ of\ Li\ element}{SF} : \frac{the\ peak\ area\ of\ F\ element}{SF} = 0.48625 : 0.51375$$

Thus, x is 1.06, indicating that the LiF_x is fully stoichiometric.

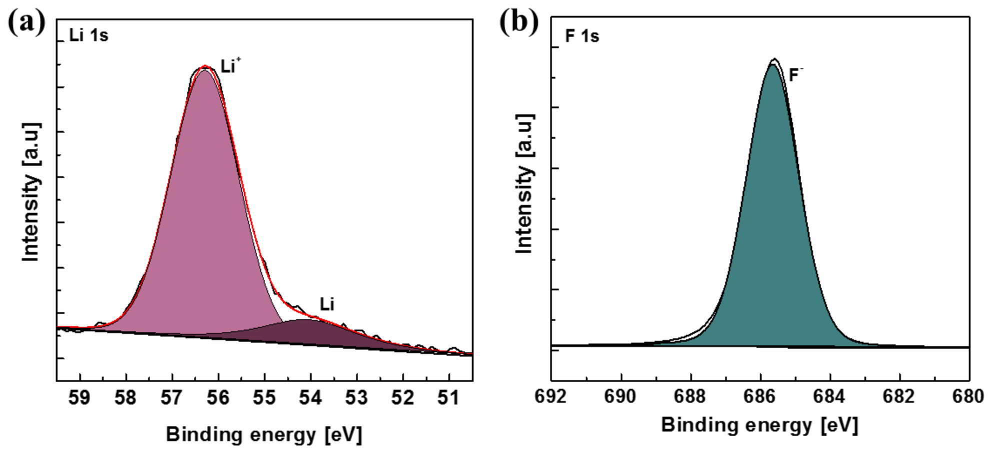

Figure S3. The core level of LiF_x film measured by XPS.

(a) Li 1s core level; (b) F 1s core level.

Figure S4:

The content curves of all elements with the etching depth from 0 to 291.9 s are summarized in the Figure S4. There is no obvious interface in the LiF_x/Mg stack, which causes a hybrid phase film composed of Mg-O, Mg-F and Mg. The existence of Mg-F, Mg-O, Li-F and Mg unambiguously proves the interchange of F, O with Mg atoms after precisely control R_{Mg} combined with low-temperature and varied vacuum post-annealing process, resulting in a transparent LiF_x/MgF_xO_y film stack.

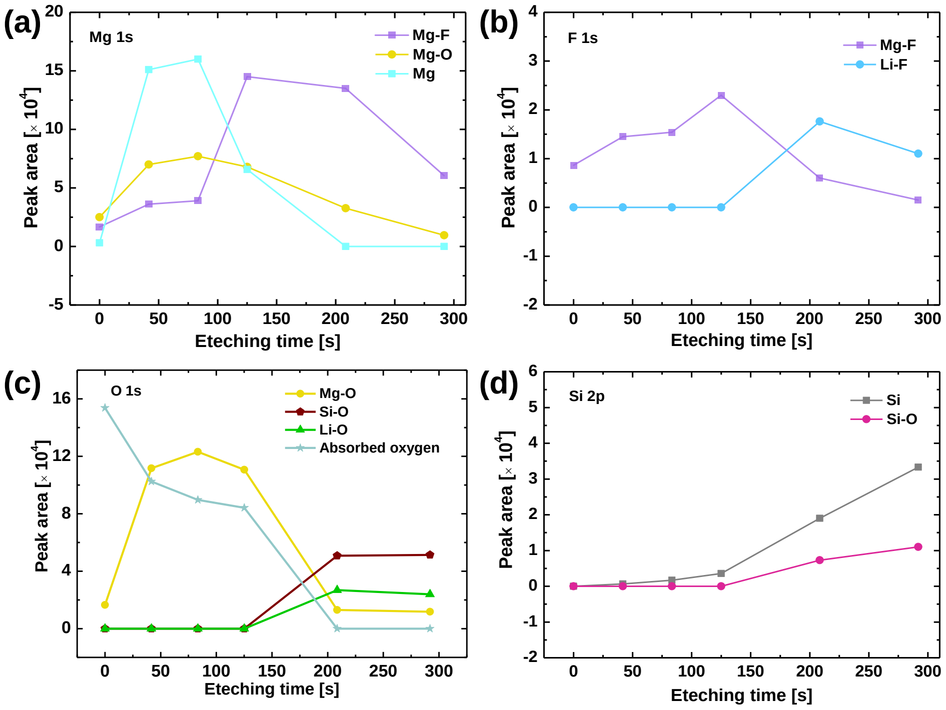

Figure S4. The elements peak area evaluation with the Ar etching time from 0 to 291.9 s. (a) Mg 1s core level; (b) F 1s core level; (c) O 1s core level; (d) Si 2p core level.

Figure S5:

Preparation of LiF_x/MgF_xO_y stacks*:* 0.75 nm LiF_x film was deposited by thermal evaporation at room temperature, followed by thermal evaporated 14 nm-thick Mg from Mg source (99.995%) on LiF_x layer at a base pressure of ~ 6.5×10^{-4} Pa with different growth rate (from < 0.1 Å/s to 0.9 ± 0.1 Å/s). Then, LiF_x/Mg stack was post-annealed for 90 min at 120 °C under a varied vacuum from 10^{-4} Pa to 10^{-1} Pa. *Characterization of* LiF_x/MgF_xO_y*:*The conductivity of LiF_x/MgF_xO_y stacks with different Mg deposition rate on a glass substrate was measured by home-made two-probe instrument (2450 SourceMeter) at room temperature.

The conductivity (σ) of LiF_x/MgF_xO_y film stack with different R_{Mg} was shown in Figure S5. The conductivity of LiF_x/MgF_xO_y at extremely low deposition rate (R_{Mg} < 0.1 Å/s) is close to that of pure MgF_x. The conductivity increases from about 1.8×10^{-5} S/cm to 2978.4 S/cm as R_{Mg} increases from 0.15 Å/s to 0.9 ± 0.1 Å/s, exhibiting adjustable conductivity. The highest conductivity of LiF_x/MgF_xO_y could be ascribed to the larger proportion of Mg in MgF_xO_y films. The lowest conductivity value may be due to majority of Mg-F and Mg-O in the MgF_xO_y film.

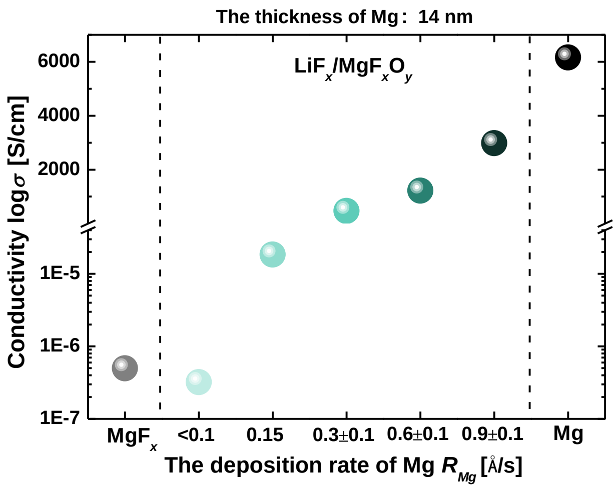

Figure S5. The conductivity of LiF_x/MgF_xO_y film stack under different Mg deposition rate (< 0.1 Å/s to 0.9 ± 0.1 Å/s), and the conductivity of pure MgF_x and Mg.

Figure S6:

Contact Measurement: Contact resistivity ρ_c of three contact structures (n-Si/i-a-Si:H and LiF_x/MgF_xO_y, n-Si/i-a-Si:H/LiF_x/MgF_xO_y and Ag, n-Si/i-a-Si:H/LiF_x/MgF_xO_y and ITO/Ag) were measured by using the Cox and Strack and transfer length method (TLM). The LiF_x/MgF_xO_y contact (LiF_x = 0.75 nm, Mg = 14 nm or 70 nm, covering layer Ag = 350 nm, respectively) were fabricated with different Mg deposition rate from < 0.1 Å/s to 0.9 ± 0.1 Å/s. Each TLM set was isolated along its edges to confine the current, the contact resistivity ρ_c was extracted by fitting the trend of resistance versus spacing of the rear contacts.

The conductivity of LiF_x/MgF_xO_y stack also influences the contact resistance (ρ_c) of LiF_x/MgFO with different R_{Mg} and structures. The Cox and Strack and transfer length method (TLM) is presented in Figure S6. The contact resistance between LiF_x/MgF_xO_y stack and i-a-Si:H exhibits the highest ρ_c (~600 mΩ·cm^2 average) when the R_{Mg} < 0.1 Å/s. The ρ_c decreases sharply to 22.4 mΩ·cm^2 with the increasing deposition rate of 0.9 ± 0.1 Å/s, and the thicker Mg film the lower contact resistance. The lowest conductivity of 2.0 mΩ·cm^2 between LiF_x/MgF_xO_y and i-a-Si:H was achieved at high R_{Mg} with 70 nm Mg. Combining the carrier transport mechanism of heterojunction structure and the conductivity results in Figure S5, we find the tunneling effect plays the significant role for the contact resistance.

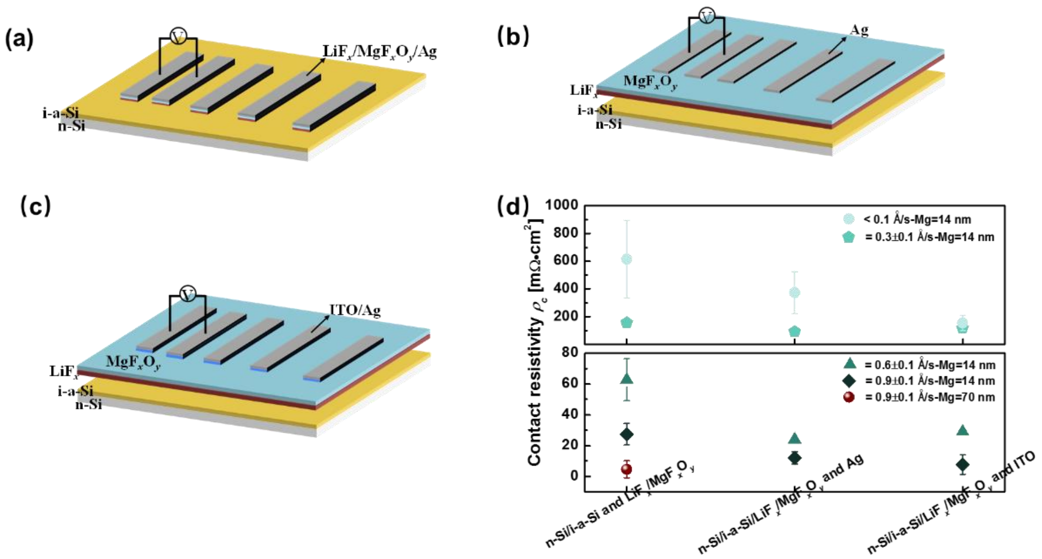

Figure S6. Depicts a schematic of the contact resistivity (ρ_c) using the Cox and Strack method. (a) n-Si/i-a-Si:H and LiF_x/MgF_xO_y; (b) n-Si/i-a-Si:H/LiF_x/MgF_xO_y and Ag; (c) n-Si/i-a-Si:H/LiF_x/MgF_xO_y and ITO/Ag; (d) The contact resistivity (ρ_c) of three structure.

Figure S7:

Preparation of LiF_x/Mg film stacks*:* 0.75 nm LiF_x film was deposited on glass substrate, followed by thermal evaporated 14 nm-thick Mg with a deposition rate (< 0.1 Å/s). Then, LiF_x/Mg was post-annealed for 90 min at 120 ^{o}C or the film stacks was not post-annealing treatment. *Characterization of* LiF_x/Mg film stacks*:* The surface morphologies of LiF_x/Mg on glass substrate with or without annealing treatment were measurement by Scanning electron microscope (SEM). In order to clearly observe the morphology, the surface of LiF_x/Mg film was sprayed with about hundred nanometers of gold.

The influence of post annealing process on surface morphology of LiF_x/Mg stack and device performance is systematically analyzed. SEM images on glass substrate of LiF_x/Mg (< 0.1 Å/s) and LiF_x/Mg (0.9 ± 0.1 Å/s) with and without 120 ^{o}C annealing are presented in Figure S7. In this work, we define LiF_x/Mg (120 ^{o}C annealing) as LiF_x/MgF_xO_y hybrid film stack. However, in order to compare the effect of post-annealing, we still employ LiF_x/Mg and LiF_x/Mg (120 ^{o}C annealing) here.

As shown in SEM images of Figure S7, the surface morphology of LiF_x/Mg stack is significantly influenced by post annealing process. It is obviously the stack film is more uniform and denser after post annealing, for both low Mg deposition rate and high Mg deposition rate. It also can be found that the grain size at high Mg deposition rate is larger. It is well known that the LiF_x and MgF_x is amorphous, the detected particles should be the existence of Mg, which may be beneficial for both contact resistivity and device performance.

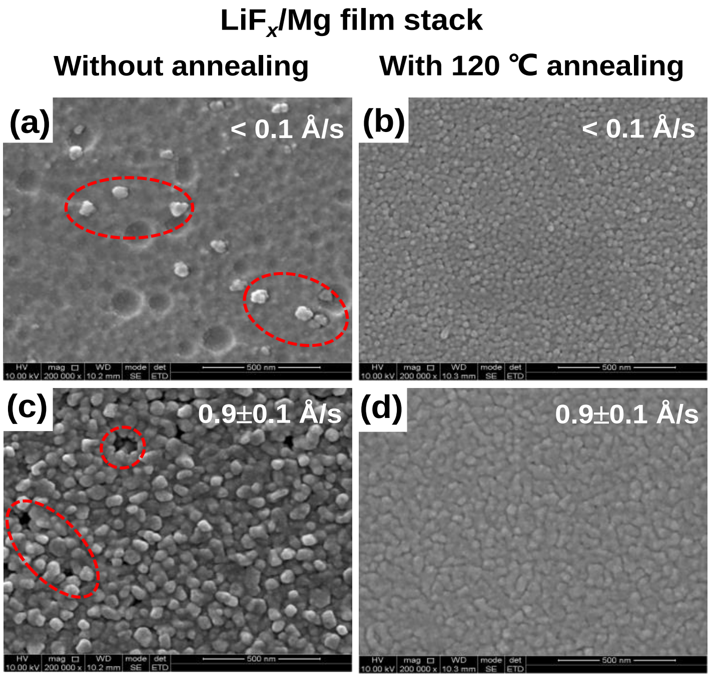

Figure S7. (a)-(d) SEM images of LiF_x/Mg (< 0.1Å/s) and LiF_x/Mg (0.9 ± 0.1 Å/s) with and without 120 °C annealing on glass substrate.

Figure S8:

The post-annealing process further improves film quality, contact characteristics and passivation effect, resulting in higher *FF*, V_{oc} and J_{sc} as shown in Figure S8. All in all, according to the results of SEM images, contact resistivity and *J-V* performances, the deposition rate of Mg plays a major role, post-annealing plays an auxiliary role and both are indispensable to realize a high-performance LiF_x/MgF_xO_y electron selective extraction stacks.

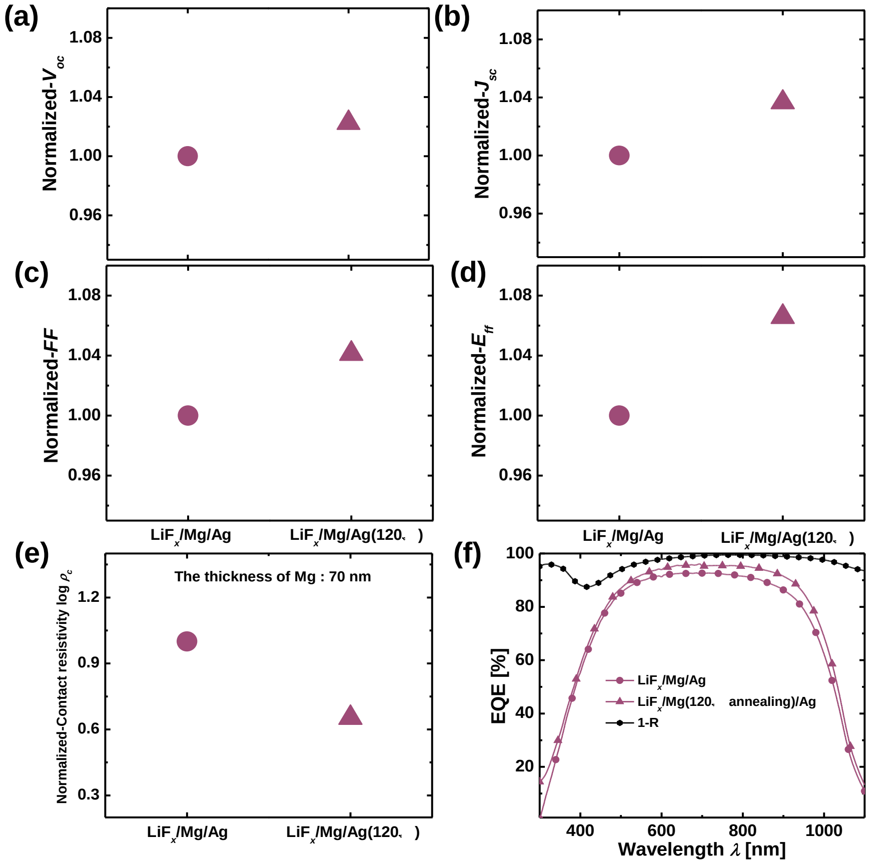

Figure S8. The detailed normalized photoelectrical parameters of dopant-free silicon solar cells with MoO_x/IZO front contact and LiF_x/Mg combined with a capping layer Ag as rear contact with and without post-annealing, respectively. (a) V_{oc}, (b) J_{sc}, (c) *FF*, (d) E_{ff} and (f) EQE. (e) The contact resistivity of LiF_x/Mg with and without post-annealing with n-Si/i-a-Si:H substrate.

Figure S9:

Preparation of MoO_x film*:* 20 nm MoO_x film was deposited on ITO-glass substrate to measure surface potential and obtain work function by thermal evaporation from a MoO_3 source (99.99%) at room temperature with a growth rate 0.1 Å/s at a base pressure of ~ 6×10^{-4} Pa.

Characterization of MoO_x film*:* The surface potential is associated with the work function (*WF*), which is determined by contact potential difference (CPD) between a Pt-coated conductive cantilever probe and the samples. $\emptyset_{tip}$ is work function of the tip, $\emptyset_{MoOx}$ is work function of the sample surface, and e is the elementary charge of electron. The work function of the Pt/Cr-coated tip was calibrated with the HOPG sample ($\emptyset_{HOPG} = 4.6\ eV, CPD = 420\ mV$).

$$\emptyset_{MoOx} = \emptyset_{tip} - eCPD_{MoOx}$$

$$\emptyset_{MoOx} = (5.02 + 0.912)eV$$

$$\emptyset_{MoOx} = 5.93\ eV$$

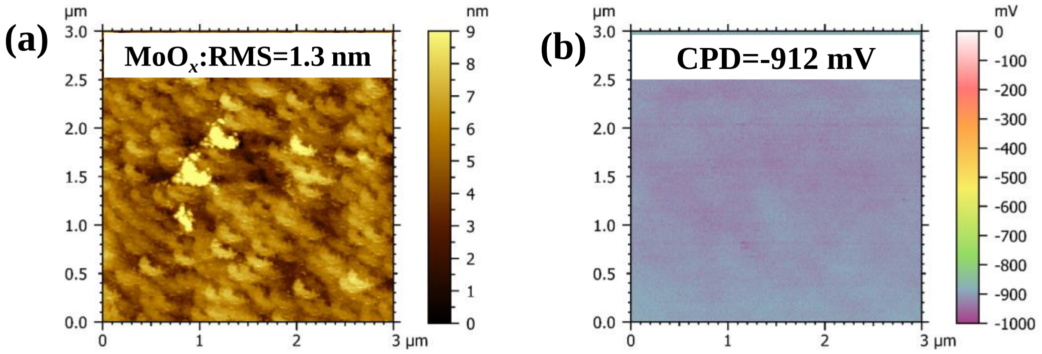

Figure S9. (a) (b) Surface morphology and surface potential distribution of MoO_x film, respectively, where RMS is root-mean-square roughness of surface.

**Electron contact structure**	E_{ff} **[%]**	V_{oc} **[mV]**	J_{sc} **[mA/cm²]**	***FF*** **[%]**
n-Si/i-a-Si/LiF_x/MgF_xO_y/ITO (80 nm)/Ag grid (front side)	16.9	691.6	37.4	65.3
n-Si/i-a-Si/LiF_x/MgF_xO_y/ITO (80 nm)/Ag grid (rear side)	12.0	683.8	27.2	64.7

Table S1. The detailed electrical parameters (V_{oc}, J_{sc}, *FF*, and E_{ff}) of dopant-free bifacial silicon solar cells of Figure 5B.

Figure S10:

Characterization of devices: The quantum efficiency of dopant-free solar cells with LiF_x/MgF_xO_y electron selective contact was measured by a system QE measurement system.

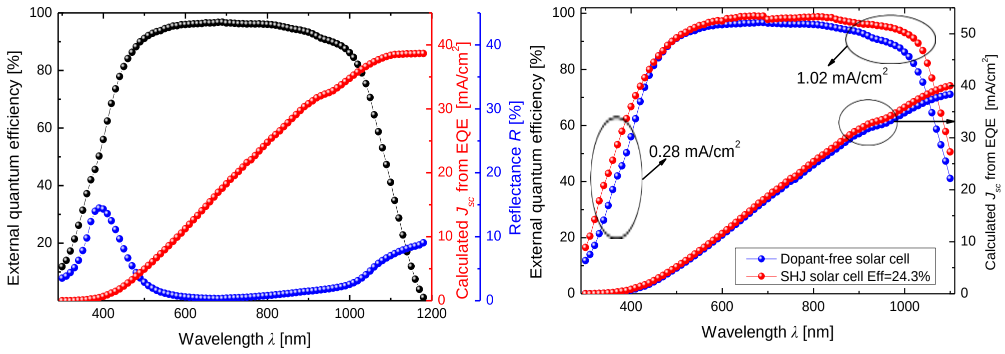

Figure S10. The external quantum efficiency (EQE) and calculate the photogenerated current density from spectral response is presented. The calculated J_{sc} from EQE of monofacial dopant-free solar cell is 38.65 mA/cm^2 in this work. Compare with EQE results of SHJ solar cell with an impressive efficiency of 24.3%, the spectral response of dopant-free solar cell in this work is obviously lower at 300-500 nm and 800-1100 nm wavelength band, resulting in a 0.28 mA/cm^2 and 1.02 mA/cm^2 photogenerated current density loss, respectively.
